# Etymologia: *Listeria*

**DOI:** 10.3201/eid2204.ET2204

**Published:** 2016-04

**Keywords:** etymologia, Listeria, bacteria, Joseph Lister

## *Listeria* [lis-teʹre-ə]

A genus of small, gram-positive, rods, *Listeria* was first isolated by Murray in 1924 as *Bacterium monocytogenes*. In 1927, Pirie proposed the genus *Listerella* in honor of British surgeon Sir Joseph Lister (1827–1912), an early advocate of antiseptic surgery. It was not until 1939 that Pirie realized that this genus had already been taken by a slime mold (also named in honor of Lister, by Jahn in 1906). In 1940, he proposed the alternative name *Listeria*. The mouthwash Listerine was also named after Lister, in 1879 by Lawrence and Bosch, when it was marketed as a surgical antiseptic.
